# Integrated investigation of the prognostic role of HLA LOH in advanced lung cancer patients with immunotherapy

**DOI:** 10.3389/fgene.2022.1066636

**Published:** 2022-12-01

**Authors:** Xiaotao Zhang, Hongzhen Tang, Haitao Luo, Huiping Lu, Chaohu Pan, Haiming Yu, Linlin Zhang, Yaping Guan, Lan Yu, Huili Chu, Jun Chen, Jun Wang

**Affiliations:** ^1^ Affiliated Qingdao Central Hospital of Qingdao University, Qingdao, China; ^2^ YuceBio Technology Co., Ltd., Shenzhen, China; ^3^ Department of Medical Oncology, Tianjin Medical University General Hospital, Tianjin, China; ^4^ Department of Oncology, The First Affiliated Hospital of Shandong First Medical University and Shandong Provincial Qianfoshan Hospital, Jinan, China; ^5^ Shandong Lung Cancer Institute, Jinan, China; ^6^ Shandong Key Laboratory of Rheumatic Disease and Translational Medicine, Jinan, China; ^7^ Department of Oncology, The 960th Hospital of PLA, Jinan, China; ^8^ Department of Lung Cancer Surgery, Tianjin Lung Cancer Institute, Tianjin Medical University General Hospital, Tianjin, China

**Keywords:** HLA, LOH, immunotherapy, PD-L1, TMB, CD8^+^ T cell, lung cancer

## Abstract

Although multiple studies have shown that loss of heterozygosity (LOH) at the human leukocyte antigen (HLA) locus is one of the mechanisms of immune escape, the effect of HLA LOH on the immunotherapy response of patients is still unclear. Based on the data of 425 Chinese lung cancer patients, the genomic characteristics with different HLA LOH statuses were analyzed. The driver genes mutation frequency, oncogenic signaling pathways mutation frequency, tumor mutational burden (TMB) and chromosomal instability (CIN) score in the HLA LOH high group was significantly higher than in the HLA LOH negative group. Transcriptome analyses revealed that pre-existing immunologically active tumor microenvironment (TME) was associated with HLA LOH negative patients. Non-small cell lung cancer (NSCLC) patients, especially for lung squamous cell carcinomas (LUSC), with HLA LOH negative have a longer survival period than those with HLA LOH. In addition, the combination of HLA LOH with TMB or programmed cell death-Ligand 1 (PD-L1) expression can further distinguish responders from non-responders. Furthermore, a comprehensive predictive model including HLA LOH status, TMB, PD-L1 expression and CD8^+^ T cells was constructed and exhibited a higher predictive value, which may improve clinical decision-making.

## Introduction

Treatment landscapes for patients with advanced cancer have been revolutionized with immune checkpoint inhibitors (ICIs) by reinvigorating one’s own T cell-mediated immune response (Le et al., 2015; Hellmann et al., 2018a). Several ICIs have been approved as the first-line or second-line or back-line treatment for multiple cancers (Herbst et al., 2016; Reck et al., 2016; Gettinger et al., 2018; Mok et al., 2019; Andre et al., 2020). However, only a small subset of patients benefits from the treatment of ICIs. Although several biomarkers such as tumor mutational burden (TMB) and programmed cell death-Ligand 1 (PD-L1) have been shown to predict the efficacy of immunotherapy, some patients with TMB-high or PD-L1-high are still resistant to immunotherapy (Giroux Leprieur et al., 2017; Hellmann et al., 2018a; Hellmann et al., 2018b; Samstein et al., 2019; Herbst et al., 2020; Montesion et al., 2021). Therefore, the development of superior predictive biomarkers and a deeper understanding of the mechanisms for resistance are urgently required.

Tumor cells are recognized by CD8^+^ T cells *via* human leukocyte antigen class I (HLA-I) which presents the tumor-specific mutant peptides (Schumacher and Schreiber, 2015). loss of heterozygosity (LOH) at the human HLA-I locus interrupts tumor antigen recognition and has been described as a potential mechanism of immune escape (McGranahan et al., 2017; Chowell et al., 2018). The previous study has shown a high incidence of HLA LOH in a variety of cancer, and TMB in patients with HLA LOH was higher (Shim et al., 2020). However, the genomic and tumor microenvironment (TME) features with different statuses of HLA LOH are unclear, especially for Chinese patients.

Recently, several studies have identified the significance of HLA LOH in immunotherapy (Chowell et al., 2018; Shim et al., 2020; Montesion et al., 2021). The response to ICIs was affected by the genotype of HLA-I alleles (Chowell et al., 2018). The predictive efficacy of ICIs was improved using the corrected TMB (Shim et al., 2020). However, the clinical implication of HLA LOH in patients treated with ICIs has not been well characterized. In addition, due to the complexity of anti-tumor mechanisms, a single marker cannot accurately distinguish responders (Wang et al., 2022). Therefore, a comprehensive predictive model is required.

In this study, targeted panel sequencing from 425 Chinese lung cancer patients was performed to investigate the genomic features between the different statuses of HLA LOH. Transcriptome data from The Cancer Genome Atlas (TCGA) were used to study the impact of HLA LOH on the TME. The predictive efficacy of HLA LOH alone and its combination with TMB or PD-L1 were analyzed. Furthermore, a comprehensive predictive model based on HLA LOH, PD-L1, TMB and CD8^+^ T cells was constructed, and its association with clinical responses to ICIs was characterized, which may help clinical decision-making in non-small cell lung cancer (NSCLC) patients treated with ICIs.

## Materials and methods

### Samples and datasets

Samples from 425 Chinese patients with lung cancer between January 2019 and June 2020 were retrospectively obtained. The inclusion criteria were as follow: 1) lung cancer, 2) performed with targeted panel sequencing and immunohistochemistry (IHC) assay for PD-L1. The clinical information of the patients is shown in Supplementary Table S1. Genomic and transcriptome data of 969 NSCLC patients from TCGA were used to analyze the difference in gene expression, enriched pathway and cell proportions across the different statuses of HLA LOH. (Thorsson et al., 2018).

Genomic and clinical data from 89 NSCLC patients treated with ICIs were obtained to analyze the predictive efficacy of HLA LOH, TMB, PD-L1 and CD8^+^ T cells on immunotherapy, and the comprehensive predictive model was constructed based on these data (Anagnostou et al., 2020). The classification criteria in the original article was used to define patients as TMB high and low. For PD-L1, the proportion of PD-L1+ cells higher than 1% was defined as PD-L1 high, and the others was defined as PD-L1 low. The cut-off values for CD8^+^ T high and CD8^+^ T low were defined as the median values of CD8^+^ T cells.

### IHC assay for PD-L1

The Dako PD-L1 IHC 22C3 pharmDx assay was used to detect PD-L1 protein expression in formalin-fixed paraffin-embedded (FFPE) tumor tissue slides according to the manufacturer’s recommendations (Kulangara et al., 2019; Lantuejoul et al., 2020). Briefly, the FFPE slides were heated at 65°C for 30 min, and then dewaxed, rehydrated, and fixed. Next, the slides were incubated with anti-PD-L1 antibody (clone 22C3). After three thorough washes, the slides were incubated with horseradish peroxidase (HRP)-conjugated secondary antibody. After incubation, the tyramide signal amplification (TSA) was added to the slides at the dilution of 1:100. Finally, the slides were stained with 4′-6′-diamidino-2-phenylindole (DAPI) at the dilution of 1:10. Images were captured with a Vectra 3.0 pathology imaging system microscope (PerkinElmer Inc.). A three-tiered grading system was applied to evaluate the proportion of PD-L1 expression in tumor cells: “negative” (Tumor Proportion Score (TPS) < 1%), “intermediate” (1% ≤ TPS <50%), and “high” (TPS ≥50%).

### Targeted panel sequencing and data analysis

Genomic DNA was isolated from tumor biopsies and matched peripheral-blood samples using the GeneRead DNA FFPE Kit (Qiagen, 180,134). All sample capture libraries were prepared using the YuceOne Plus v2.2 (YuceBio, Shenzhen, China). The target capture specific probe is hybridized with genomic DNA to enrich the DNA fragments of the target genomic region and then sequenced using high-throughput sequencing technology. SOAPnuke (v1.5.6) was used to filter the original reads to remove low-quality reads with the unknown base “N” greater than 10% (Chen et al., 2018). BWA (v0.7.12) was used to align the clean reads with the human reference genome (UCSC GRCh37/hg19) (Li and Durbin, 2009). Somatic mutations were detected with the VarScan (Version 2.4) (Koboldt et al., 2012). Possible false-positive mutations were filtered using Bcftools (1.14) with the parameter set as follow: “basicfilter = """' (STRLEN (REF) > 50 || STRLEN (ALT) > 50) || INFO/STATUS!∼"Somatic"'""" hotspotfilter = """'INFO/HOTSPOT! = "." && ((INFO/SOR! = 0 && INFO/SOR<3) || INFO/VD < 5 || INFO/AF<0.007 || INFO/SSF>0.05)'""" fpdbfilter = """'INFO/HOTSPOT = "." && ((INFO/FPDB! = "0" && INFO/FPDB! = ".") || (INFO/GERMLINE! = "0" && INFO/GERMLINE! = "."))'""" normalfilter = """'INFO/HOTSPOT = "." && ((INFO/GERMLINE)! = "." || (FORMAT/PMEAN [0]<20)||((INFO/SOR! = 0 && INFO/SOR<5) || INFO/AF<0.02 || INFO/SSF>0.01)||(INFO/AF<0.05 && FORMAT/MQ [0]<50)||(FORMAT/MQ [0]<30)||(INFO/AF<0.05 && FORMAT/QUAL [0]<30) || ((INFO/MSI>10||(INFO/MSILEN>1 && INFO/MSI>4)) && INFO/AF<0.3)||(type! = "snp” && INFO/MSI>3 && ((INFO/MSILEN=(strlen (REF)-1))||(INFO/MSILEN=(strlen (ALT [0])-1))) && INFO/AF<0.1) || (FORMAT/NM [0]>2 && FORMAT/MQ [0]<60 && INFO/AF<0.2) || (FORMAT/NM [0]>3 && (FORMAT/MQ [0]<55||FORMAT/NM (Hellmann et al., 2018a)>3)) || (FORMAT/DP [0]<30 || FORMAT/DP (Hellmann et al., 2018a)<30)|| INFO/VD < 10 || (FORMAT/BIAS [0:0] = "2" && FORMAT/BIAS [0:1] = "1") || (FORMAT/SBF [0] < 0.05 && FORMAT/VD [0]<50) || ((INFO/SOR! = 0 && INFO/SOR<10) && FORMAT/MQ [0]<60))' """“. Identified somatic mutations were annotated with SnpEff (Version 4.3) (Cingolani et al., 2012). Somatic copy number alterations (SCNAs) were analyzed using Allele-Specific Copy number Analysis of Tumors (ASCAT) (v3.1.0) with default parameters (Van Loo et al., 2010).

### HLA typing and LOH analysis

HLA-I typing of tumors and matched normal samples was performed as previously described (Yi et al., 2021). In short, the patched opitype software was used for HLA typing (Szolek et al., 2014). Then loss of heterozygosity in human leukocyte antigen (LOHHLA) algorithm was performed to identify HLA LOH in tumor samples (McGranahan et al., 2017). It is classified as LOH if the following two conditions are met: 1) A copy number <0.5 and 2) allelic imbalance is determined with *p* < 0.01 using the paired Student’s *t*-Test between the two distributions. According to the proportion of LOH in HLA alleles, LOH status was further divided into three grades: negative (all HLA alleles had no LOH), low (LOH/HLA alleles are 1/6, 2/6, or 1/5), high (LOH/HLA alleles are 1/3, 1/4, ≥3/6 or ≥2/5).

### Genomic biomarker calculation and gene mutation pathway analysis

TMB was determined as the number of non-synonymous mutations per megabase of the genome examined. All nonsynonymous mutations and their upstream and downstream nucleotide sequences of 10 amino acids were selected and translated into 21-mer peptides. The translated peptide was used to produce 8- to 11-mer peptides with a sliding window approach. The binding affinity of peptide and major histocompatibility complex (MHC) class I was predicted by NetMHCpan (version 3.0) (Hoof et al., 2009). If the predicted half-maximum inhibitory concentration (IC50) binding affinity was not greater than 500 nM, the peptide was selected. Multiple selected peptides generated by the same mutation were counted as a neoantigen. Tumor neoantigen burden (TNB) was measured as the number of such peptides per megabase of the genome examined. The chromosomal instability (CIN) score was used to estimate copy number burden, which was calculated as follows: the ploidy of the sample was generated by the ASCAT algorithm (v3.1.0). For each of the 22 autosomal chromosomes, the percentage of gained and lost genomic material was calculated relative to the ploidy of the sample. The CIN score of a sample was defined as the average of this percentage value over the 22 autosomal chromosomes (Braun et al., 2020). Gene mutation in pathways analysis was compared with the previously reported gene list (Sanchez-Vega et al., 2018).

### Gene expression and infiltration abundance of immune cells analysis

The normalized counts matrix was used to identify differentially expressed genes (DEGs) with edgeR package (Chen et al., 2016). DEGs with |log2FoldChange| > 1 and a *p*-value < 0.05 were considered as significant DEGs. The Matplotlib (v3.4.3) package was used to generate volcano plots. Kyoto Encyclopedia of Genes and Genomes (KEGG) pathway, Canonical Pathways, WikiPathways, GO Biological Processes and Reactome Gene Sets enrichment were performed with Metascape web tool with a *p*-value < 0.05 (Zhou et al., 2019). The gene expression matrix was used to estimate the level of 64 cells with xCell (Aran et al., 2017).

### Construction the comprehensive predictive model

The decision-tree was evaluated to construct the comprehensive predictive model with biomarkers of CD8^+^ T cells, TMB, PD-L1 and HLA LOH to predict the efficacy of ICIs in NSCLC patients. The predictive efficacy of the model was analyzed with the leave-one-out method. Different depth of the tree, from 3 to 20, were tested to calculate the F1 score of the comprehensive predictive model. The most optimal depth of decision tree was determined based on the highest calculated F1 score.

### Statistical analysis

Correlations between HLA LOH and clinical characteristics were analyzed using the Fisher’s exact test for categorical variables. Kruskal–Wallis rank-sum tests were used for comparisons of continuous variables across multiple groups. The *t*-test was used to compare differences between groups. Receiver operating characteristic (ROC) curve analysis was performed to measure the discriminatory ability of HLA LOH, TMB, PD-L1 and CD8^+^ T cells. Kaplan-Meier curve, Log-rank test, and cox-regression were used to assess the association of HLA LOH, TMB, PD-L1 and CD8^+^ T cells with patient survival. *p* < 0.05 was considered significant. Statistical analyses were performed in the R (v3.6.1) and Python (v3.8.8). The packages in Python were used in the study including lifelines (v0.27.0) and scipy (v1.7.3).

## Results

### Genomics characteristics of Chinese lung patients with different HLA LOH statuses

To examine tumor genomic characteristics and their potential association with different HLA LOH statuses, samples from 425 Chinese lung cancer patients were retrospectively collected and subjected to targeted panel sequencing and PD-L1 expression detection. According to the different statuses of HLA class I alleles, which are encoded by three genes (HLA-A, HLA-B, and HLA-C), 425 patients were divided into three groups: HLA LOH negative group, HLA LOH low group and HLA LOH high group. Compared with the HLA LOH negative and HLA LOH low groups, there was a significant gender discrepancy in the HLA LOH high group [68% (Male) *versus* 32% (Female)] (Supplementary Table S2).

**FIGURE 1:**
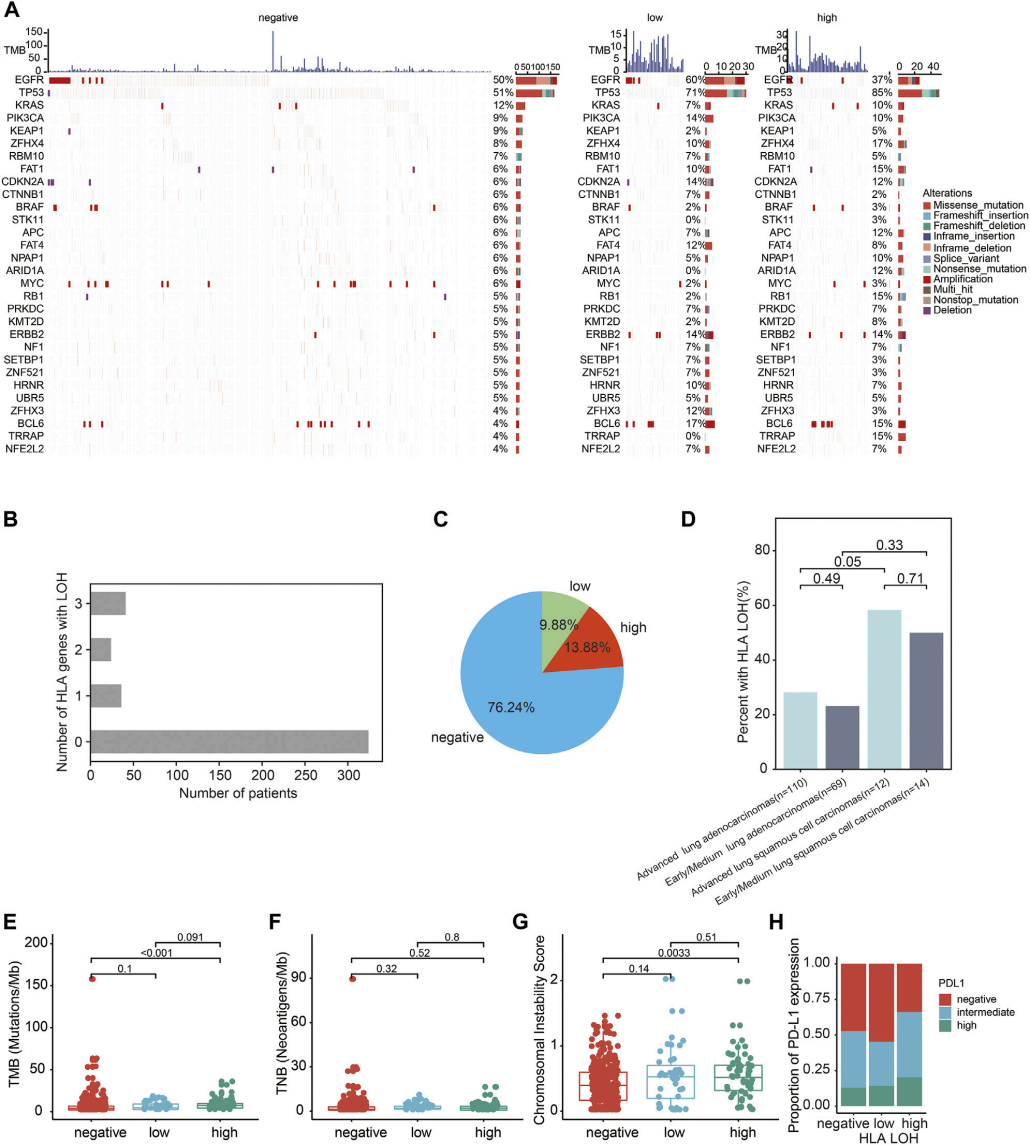
Genomics characteristics of Chinese lung patients with different HLA LOH statuses. **(A)** Genomics landscape of tumor with HLA LOH negative, HLA LOH low and HLA LOH high. The top histogram was the value of TMB. The Center heatmap is the distribution of the top 30 non-synonymous driver mutation events from patients. **(B)** The number of patients with HLA LOH negative, HLA LOH low and HLA LOH high in 425 lung cancer patients **(C)** The percentage of HLA LOH negative, HLA LOH low and HLA LOH high in 425 lung cancer patients. **(D)** The occurrence of HLA LOH in the NSCLC patients. **(E–H)** Associations between HLA LOH and TMB **(E)**, TNB **(F)**, CIN **(G)** and PD-L1 expression **(H)**.

The genomic landscapes with different statuses of HLA LOH were shown in Figure 1A. The top 10 genes were listed in order of mutated frequency: *EGFR, TP53, KRAS, PIK3CA, KEAP1, ZFHX4, RBM10, FAT1, CDKN2A, and CTNNB1* (Figure 1A)*,* which had a slight difference with the TCGA database (Cancer Genome Atlas Research, 2014). In the TCGA dataset, the top five genes with the highest mutation frequency were *TP53* (46%), *KRAS* (33%), *KEAP1* (17%), *SKT11* (17%) and *EGFR* (14%). Interestingly, a high frequency of *BCL6* amplification was found in the HLA LOH subgroup (the proportion in the HLA LOH negative, HLA LOH low and HLA LOH high groups were 4%, 17%, and 15%, respectively). *BCL6* is related to the stress response in breast cancer, lung cancer, glioma, and other solid tumors. Overexpression of *BCL6* is associated with tumor immune surveillance and drug resistance during the process of chemotherapy and radiotherapy (Fernando et al., 2019).

Among 425 lung cancer patients, the proportions of HLA LOH negative, HLA LOH low and HLA LOH high were 76.24% (324/425), 9.88% (42/425) and 13.88% (59/425), respectively. (Figures 1B,C and Supplementary Table S1). The frequency of HLA LOH in lung adenocarcinomas (LUAD) was lower than in lung squamous cell carcinomas (LUSC) (Figure 1D), which was consistent with previous studies (Zhao et al., 2021).

Furthermore, the association of genomic features and PD-L1 expression with the different statuses of HLA LOH were analyzed. It was found that TMB was significantly higher in the HLA LOH high group than in the other groups (*p* < 0.0001) (Figure 1E, Supplementary Table S2). However, there was no significant difference in TNB among these groups (Figure 1F, Supplementary Table S2). In addition, the CIN score was significantly different between the HLA LOH high and HLA LOH negative groups (*p* = 0.0033; Figure 1G). Consistent with the previous reports, the HLA LOH was related to PD-L1 expression among the three groups (*p* = 0.07642) (McGranahan et al., 2017; Montesion et al., 2021) (Figure 1H, Supplementary Table S2).

### Association of HLA LOH status with individual genes and pathways alterations

**FIGURE 2:**
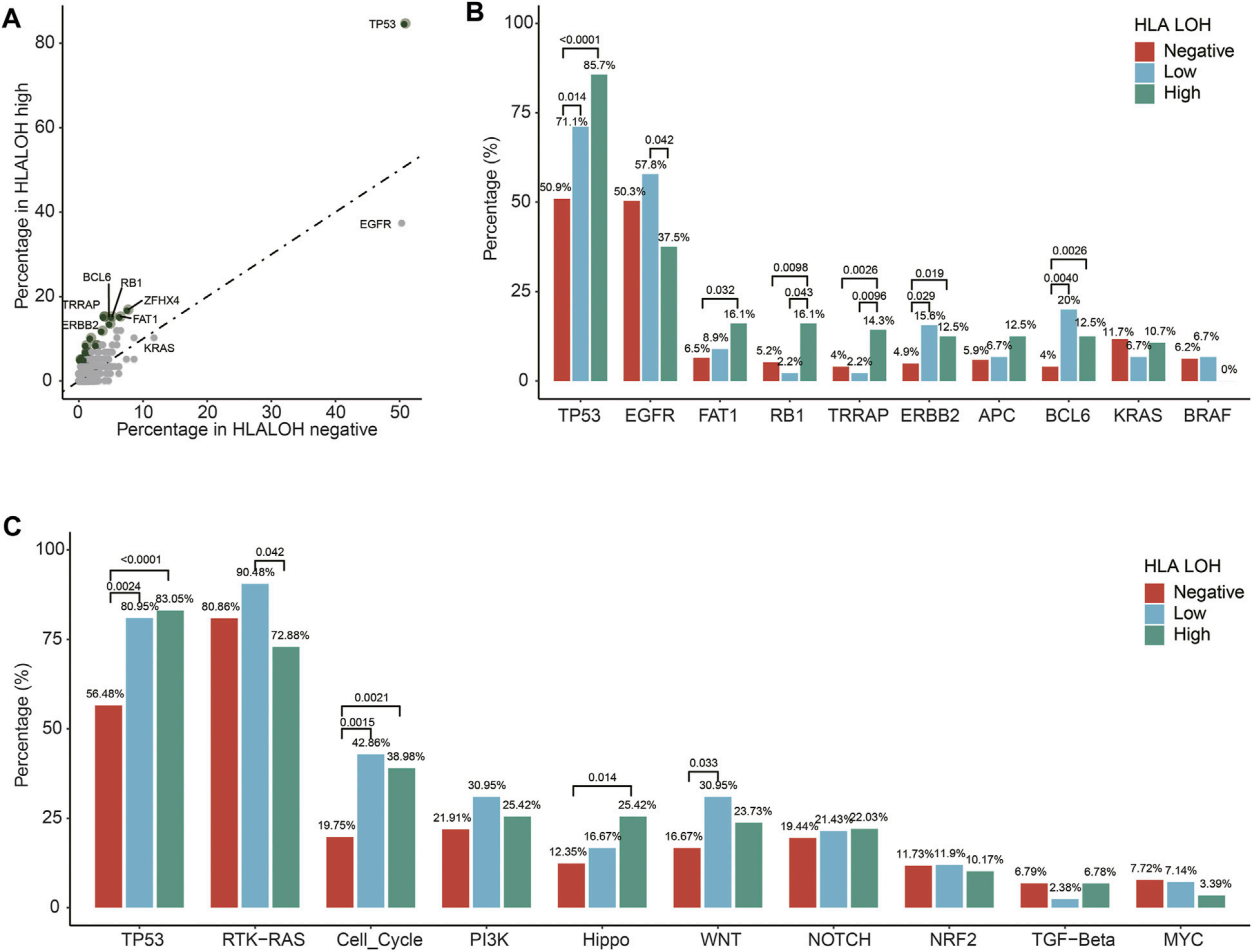
Different HLA LOH statuses based on alterations in individual genes and pathways. **(A)** Percentages of altered individual genes within HLA LOH high *versus* HLA LOH negative subgroups. Green dots denote genes associated with significantly differential HLA LOH statuses (*p*-value < 0.05) **(B)** Distribution of HLA LOH status by commonly altered genes in lung cancer. **(C)** Percentage of tumors harboring an alteration of individual pathways within HLA LOH subgroups.

To further investigate the association of gene alteration with different statuses of HLA LOH in Chinese lung cancer patients, a comparison analysis between HLA LOH high and HLA LOH negative groups was conducted. It was found that the mutation frequency of 25 genes was significantly higher in the HLA LOH high group than in the HLA LOH negative group (Figure 2A), such as the *TP53* mutations (85.7% *versus* 50.9%; *p* < 0.0001)), *FAT1* mutations (16.1% *versus* 6.5%; *p* = 0.032), *RB1* mutations (16.1% *versus* 5.2%; *p* = 0.0098), *TRRAP* mutations (14.3% *versus* 4%; *p* = 0.0026), *ERBB2* mutations (12.5% *versus* 4.9%; *p* = 0.019) and *BCL6* amplification (12.5% *versus* 4%; *p* = 0.0026) (Figure 2B). Conversely, *EGFR* mutation was associated with HLA LOH negative group when compared with HLA LOH high group (50.3% *versus* 37.5%, *p* = 0.0677). Other lung cancer-related genes, such as *APC, KRAS, and BRAF,* did not show significant differences among the groups (Figure 2B). Furthermore, the mutation in oncogenic signaling pathways were analyzed. It was found that several pathways were significantly different among the groups, such as the TP53 pathway, cell cycle pathway, Hippo pathway, WNT pathway and the RTK-RAS pathway (Figure 2C).

### Pre-existing immune programs in HLA LOH negative patients

**FIGURE 3:**
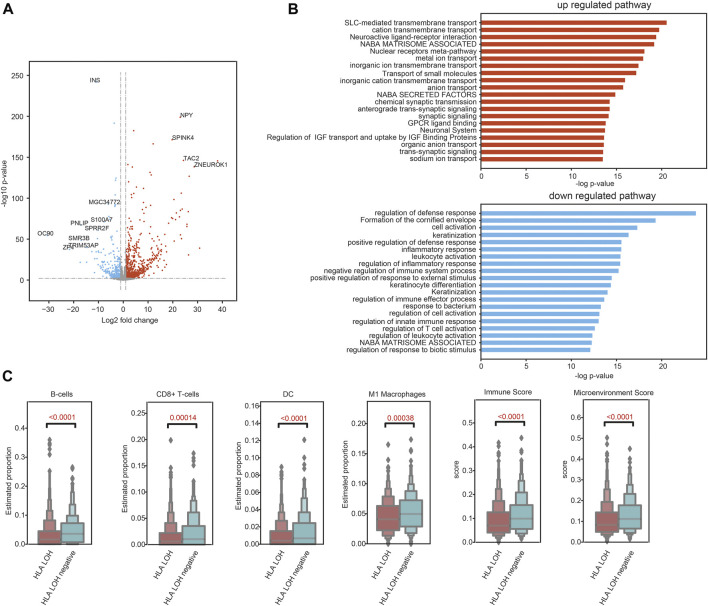
Pre-existing immune programs in HLA LOH negative patients**. (A)** Volcano plot showing DEGs between the statuses of HLA LOH and HLA LOH negative. **(B)** Function enrichment analysis for genes significantly upregulated and downregulated in HLA LOH vs. HLA LOH negative. **(C)** Comparison of the estimated proportion of lymphocytes between the statuses of HLA LOH and HLA LOH negative.

To explore the TME characteristics of patients with different HLA LOH statuses, genomic and transcriptional data of 969 NSCLC patients were obtained from TCGA. The HLA LOH was defined as the copy number of HLA class I or II-related genes less than 2. The differentially expressed genes (DEGs) between HLA LOH and HLA LOH negative groups were analyzed. There were 1081 genes significantly upregulated and 497 genes significantly downregulated in the HLA LOH groups when compared with HLA LOH negative groups (Figure 3A). Downregulated genes are associated with immunity, which suggests the pre-existing immune recognition of the tumor in HLA LOH negative patients (Figure 3B). To further verify this phenomenon, abundance of immune cells was evaluated by xCell. It was found that the proportion of B cells, CD8^+^ T cells, DC and M1 macrophages as well as the immune score and microenvironment score were significantly higher in HLA LOH negative patients when compared with HLA LOH patients. (Figure 3C). These results suggested that a pre-existing immunologically active TME may be related to HLA LOH negative patients.

### The predictive value of HLA LOH for the treatment of immunotherapy

**FIGURE 4:**
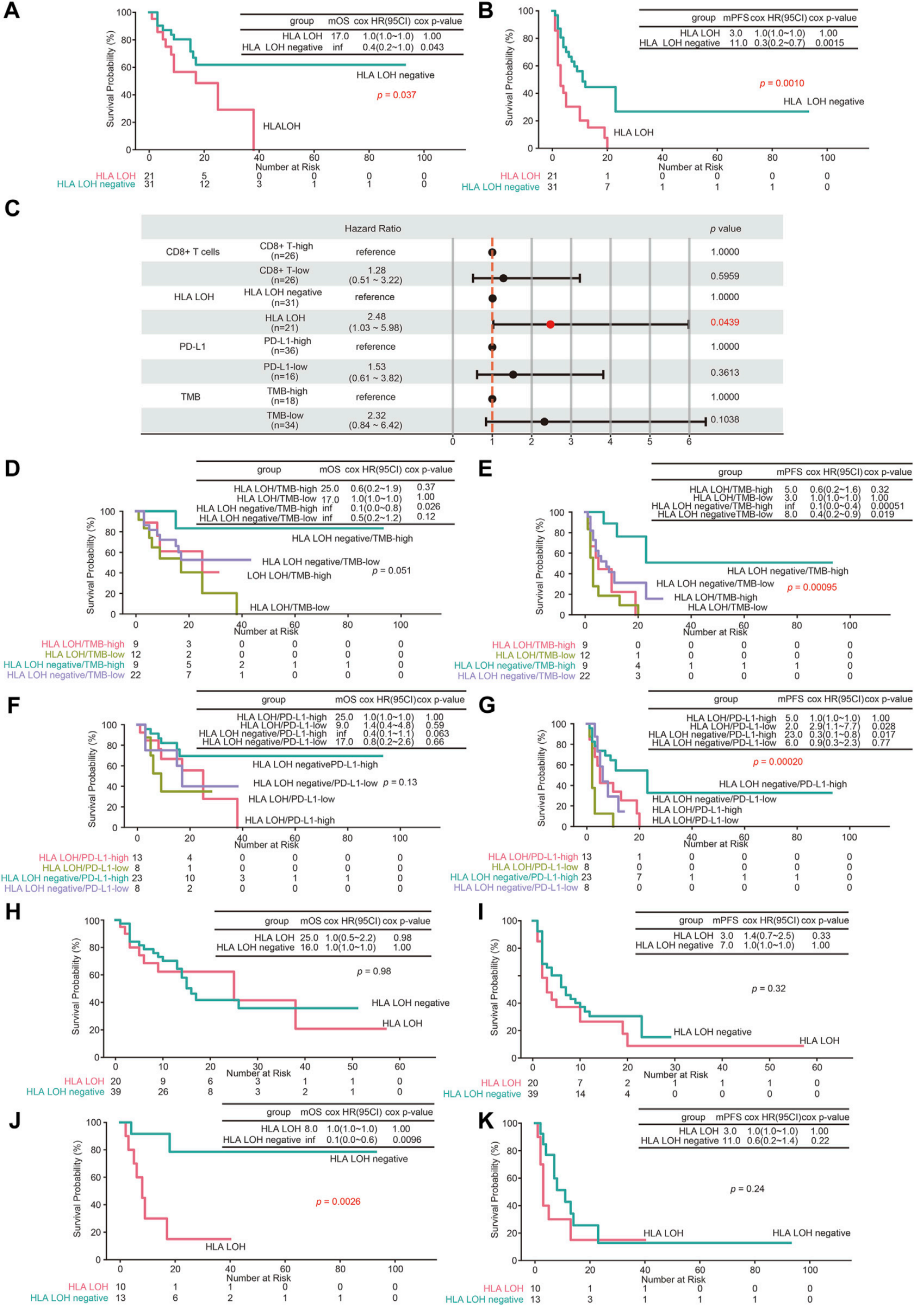
The predictive value of HLA LOH for ICIs treatment. **(A**,**B)** Kaplan–Meier curves of OS **(A)** and PFS **(B)** comparing HLA LOH negative with HLA LOH **(C)** The multivariate cox regression analyses of the HLA LOH, TMB, PD-L1and CD8^+^ T cells. **(D**,**E)** Kaplan–Meier curves of OS **(D)** and PFS **(E)** comparing groups of HLA LOH negative/TMB-high, HLA LOH negative/TMB-low, HLA LOH/TMB-high and HLA LOH/TMB-low **(F**,**G)** Kaplan–Meier curves of OS **(F)** and PFS **(G)** comparing groups of HLA LOH negative/PD-L1-high, HLA LOH negative/PD-L1-low, HLA LOH/PD-L1-high and HLA LOH/PD-L1-low **(H**,**I)** Kaplan–Meier curves of OS **(H)** and PFS **(I)** comparing HLA LOH negative with HLA LOH in LUAD patients **(J**,**K)** Kaplan–Meier curves of OS **(J)** and PFS **(K)** comparing HLA LOH negative with HLA LOH in LUSC patients.

To investigate the predictive efficacy of HLA LOH on the treatment of ICIs, genomic and clinical data of 89 NSCLC patients treated with ICIs were obtained (Supplementary Table S3). As shown in Figures 4A,B, the median overall survival (mOS) and median progression-free survival (mPFS) of patients with HLA LOH were 17 and 3 months, respectively. For patients with HLA LOH negative, the mOS was not yet reached, and the mPFS was 11 months. Patients with HLA LOH negative had a longer survival period than those with HLA LOH, which indicated that HLA LOH has a negative effect on the prognosis and may represent a mechanism of tolerance to immunotherapy. Compared with HLA LOH, it was found that TMB, PD-L1 and CD8^+^ T cell were insufficient to identify responders (Supplementary Figures S1A–F). Furthermore, multivariate cox regression was analyzed. And the result showed that only HLA LOH was significantly associated with the prognosis (Figure 4C).

TMB and PD-L1 expression were higher in HLA LOH high group (Figures 1E–H). To further investigate the prognostic association of HLA LOH with TMB and PD-L1 expression, additional stratification of HLA LOH for TMB or PD-L1 was analyzed. As shown in Figures 4D–G, the combination of HLA LOH with TMB or PD-L1 could further distinguish responders.

Compared with LUAD, the frequency of HLA LOH in LUSC was higher, which suggested that HLA LOH may have different effects on the efficacy of ICIs in LUAD and LUSC (Zhao et al., 2021). Therefore, the predictive effect of HLA LOH on immunotherapy in NSCLC with different pathological classifications was further analyzed. As shown in Figures 4H–K, HLA LOH was significantly correlated with the survival period in LUSC patients.

### The predictive efficacy of the constructed comprehensive predictive model was better than a single biomarker

**FIGURE 5:**
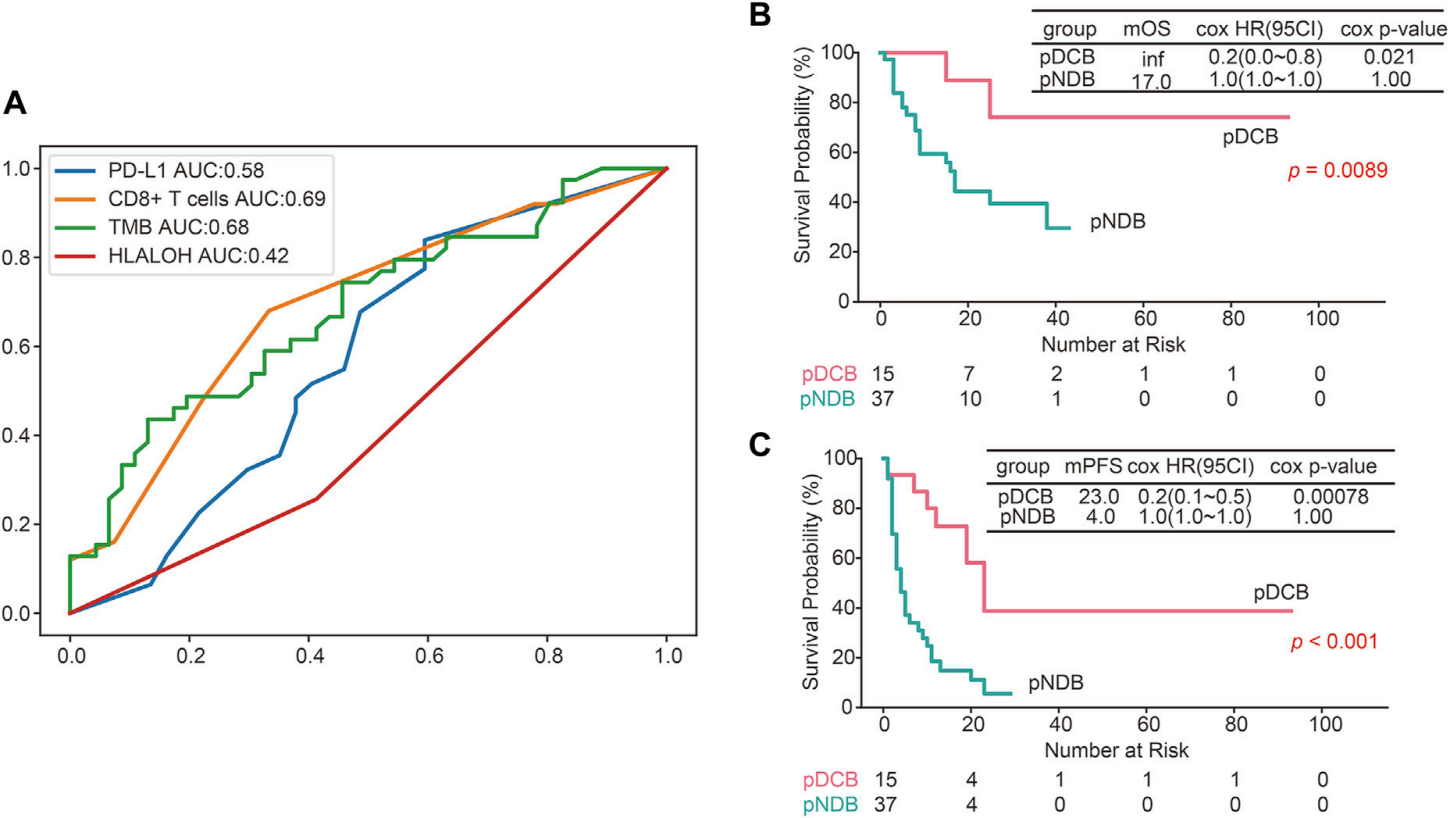
The predictive efficacy of the constructed comprehensive predictive model was better than a single biomarker. **(A)** ROC curves for PD-L1, CD8^+^ T cells, TMB and HLA LOH. **(B**,**C)** Kaplan–Meier survival curves of OS **(B)** and PFS **(C)** comparing pDCB with pNDB in NSCLC patients.

To further evaluate the predictive ability of HLA LOH for ICIs response, the ROC was plotted. As shown in Figure 5A, the area under the curve (AUC) value of HLA LOH was 0.42, indicating that HLA LOH was insufficient to distinguish responders from non-responders who were subjected to immunotherapy. Then other reported biomarkers, such as TMB, PD-L1 expression and CD8^+^ T cells were analyzed; however, none of the AUC values for these biomarkers exceeded 0.7, which suggested that a single biomarker was not effective enough to precisely distinguish patients who would benefit from the treatment of immunotherapy. In order to further improve the predictive efficacy, a comprehensive predictive model containing multiple factors related to anti-tumor immunity (HLA LOH, TMB, PD-L1 and CD8^+^ T cells) was constructed. As shown in Supplementary Figures S2A,B, the model had the highest F1 score when the decision tree depth was three. Therefore, the parameter was used to construct the comprehensive predictive model. Furthermore, the responders predicted by the comprehensive predictive model (pDCB) had a significantly longer survival period than non-responders predicted by the comprehensive predictive model (pNDB) (Figures 5B,C), and the predictive ability was better than the single biomarker (Figures 4A,B, Supplementary Figures S1A–F).

## Discussion

HLA LOH has been considered as one of the mechanisms by which tumors escape the recognition of the immune system, ultimately affecting the efficacy of immunotherapy (McGranahan et al., 2017; Chowell et al., 2018). However, the predictive efficacy of HLA LOH for immunotherapy needs to be further investigated. In this study, we found that TMB, CIN and mutation frequency of oncogene *TP53*, *ZFHX4*, *TRRAP*, *RB1*, *ERBB2* and *FAT1* were associated with the HLA LOH high group. In addition, the TME was more active in patients with HLA LOH negative than those with HLA LOH. After the treatment of ICIs, the survival period was longer in NSCLC patients with HLA LOH negative than in those with HLA LOH, especially for LUSC patients. For patients with TMB-High or PD-L1-High, the status of HLA LOH can further distinguish responders. Moreover, a comprehensive predictive model including multiple features was constructed and showed a better performance than a single biomarker.

When HLA LOH occurs, tumor cells cannot be recognized by T lymphocytes, resulting in the accumulation of TMB (Shim et al., 2020). This phenomenon has been observed in our and other research and may be the reason why some patients with TMB high remain resistant to immunotherapy. Therefore, in clinical practice, the level of TMB should be calculated along with the occurrence of HLA LOH when predicting the response to ICIs. Compared with other oncogenes, such as *TP53*, *FAT1*, *RB1*, *TRRAP*, *ERBB1* and *BCL6*, the mutation percentage of *EGFR* was lower in the HLA LOH-High group, which was similar to the previous study (Montesion et al., 2021). This result seems to suggest that HLA LOH may be a possible mechanism of resistance to immunotherapy in patients without *EGFR* mutation.

Wu et al. has revealed that the TME patterns were different in two tumors from the same patient. The tumor with HLA LOH negative showed more infiltrating CD8^+^ T cells (Wu et al., 2020). In our study, the immune-related pathways were associated with HLA LOH negative patients. In addition, antitumor lymphocytic infiltrating cells, such as CD8^+^ T cells (*p* = 0.00014), M1 macrophages (*p* = 0.00038) and DC (*p* < 0.0001) were significantly higher in patients with HLA LOH negative, indicating a more active TME.

Shim et al. and Anagnostou et al. have indicated HLA LOH cannot predict the efficacy of ICIs (Anagnostou et al., 2020; Shim et al., 2020), while Montesion et al. found there was association between HLA LOH and response to the treatment of ICIs (Montesion et al., 2021). Therefore, the predictive efficacy of HLA LOH in immunotherapy needs to be further investigated. In our study, patients with HLA LOH negative have a longer survival period than those with HLA LOH. Furthermore, the association of HLA LOH with response to ICIs in LUAD and LUSC were examined. It was found there were significant differences in survival between different HLA LOH statuses in LUSC, but not in LUAD.

There were several limitations in our study. First, there was no prognostic information in 425 Chinese lung cancer patients. Therefore, the impact of HLA LOH on prognosis cannot be assessed. Second, since there were no other cohorts simultaneously containing data of HLA LOH, CD8^+^ T cells, TMB and PD-L1, more research is needed to further verify our comprehensive predictive model. Third, therapies of ICIs combined with chemotherapy have been approved as first-line treatment schemes for NSCLC patients, we regret that there was no access to obtain clinical and multi-omics data of patients treated with ICIs combined with chemotherapy from the public dataset. Therefore, the predictive efficacy of the constructed comprehensive predictive model in patients treated with ICIs combined with chemotherapy could not be validated. Further studies are needed to investigate this comprehensive predictive model.

## Data Availability

The data presented in the study are deposited in the Genome Variation Map (GVM) repository, via the following link: https://www.cncb.ac.cn/. The accession number is PRJCA011164.

## Supplementary material

SUPPLEMENTARY FIGURE S1 Prognostic role of TMB, PD-L1 expression and CD8+ T cells in NSCLC patients treated with ICIs. **(A,B) **Kaplan–Meier curves of OS **(A)** and PFS **(B)** comparing TMB-high with TMB-low. **(C,D)** Kaplan–Meier curves of OS **(C)** and PFS **(D)** comparing PD-L1-high with PD-L1-low. **(E,F)** Kaplan–Meier curves of OS **(E)** and PFS **(F)** comparing CD8+ T-high with CD8+ T-low.

SUPPLEMENTARY FIGURE S2 Decision tree of the constructed comprehensive predictive model. **(A)** The decision tree trained with features of HLA LOH, TMB, PD-L1 and CD8+ T cells. **(B)** A heatmap of accuracy, precision, recall and f1 score for depth of decision tree from 3 to 20.
