# Supplementary figures and images for: Integrated investigation of the prognostic role of HLA LOH in advanced lung cancer patients with immunotherapy
Source: Front Genet. 2022 Dec 1;13:1066636. doi: 10.3389/fgene.2022.1066636 (PMC9751360; doi:10.3389/fgene.2022.1066636)

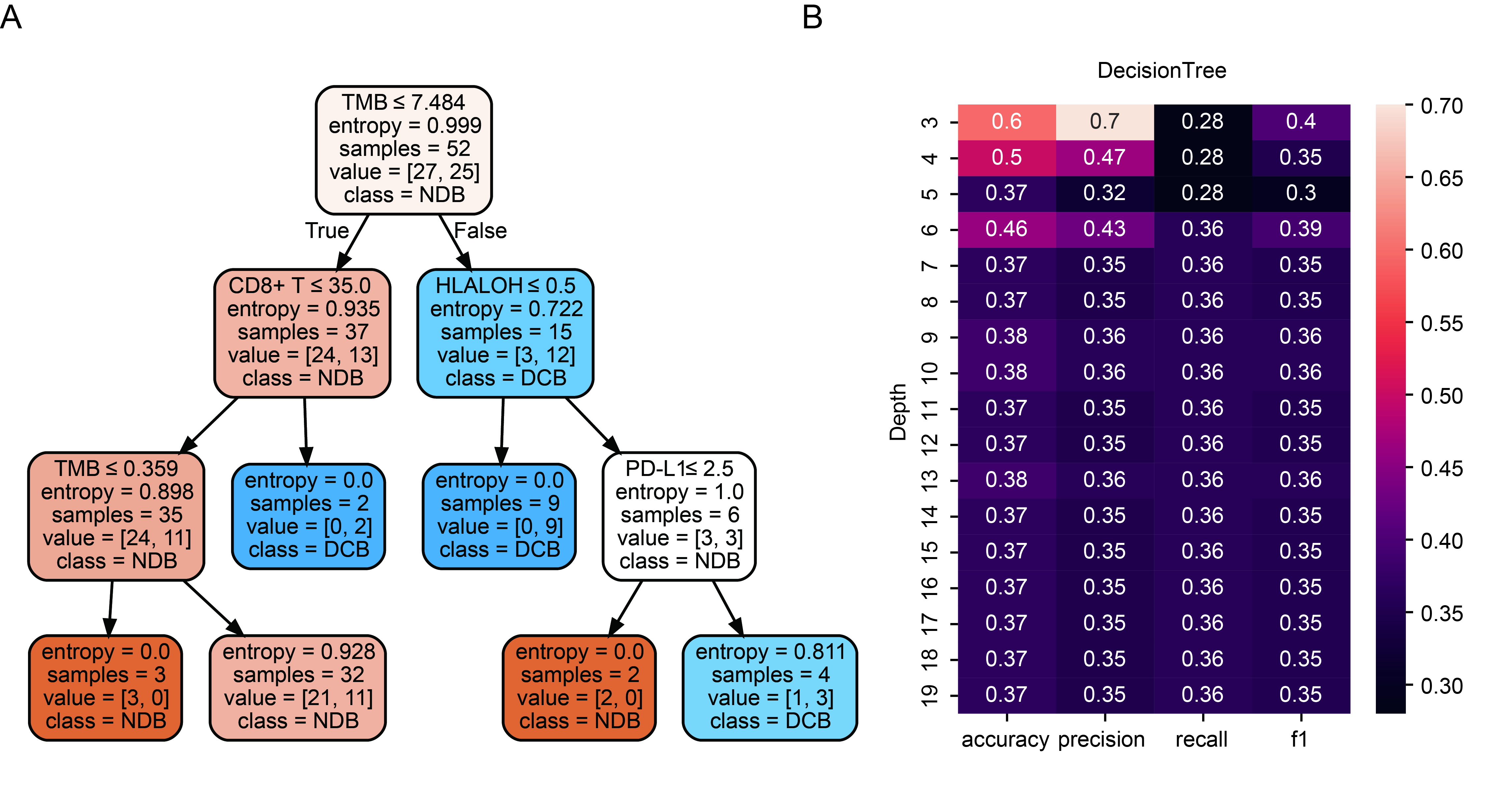

Supplement: Supplementary file 3 [file Image2.JPEG]

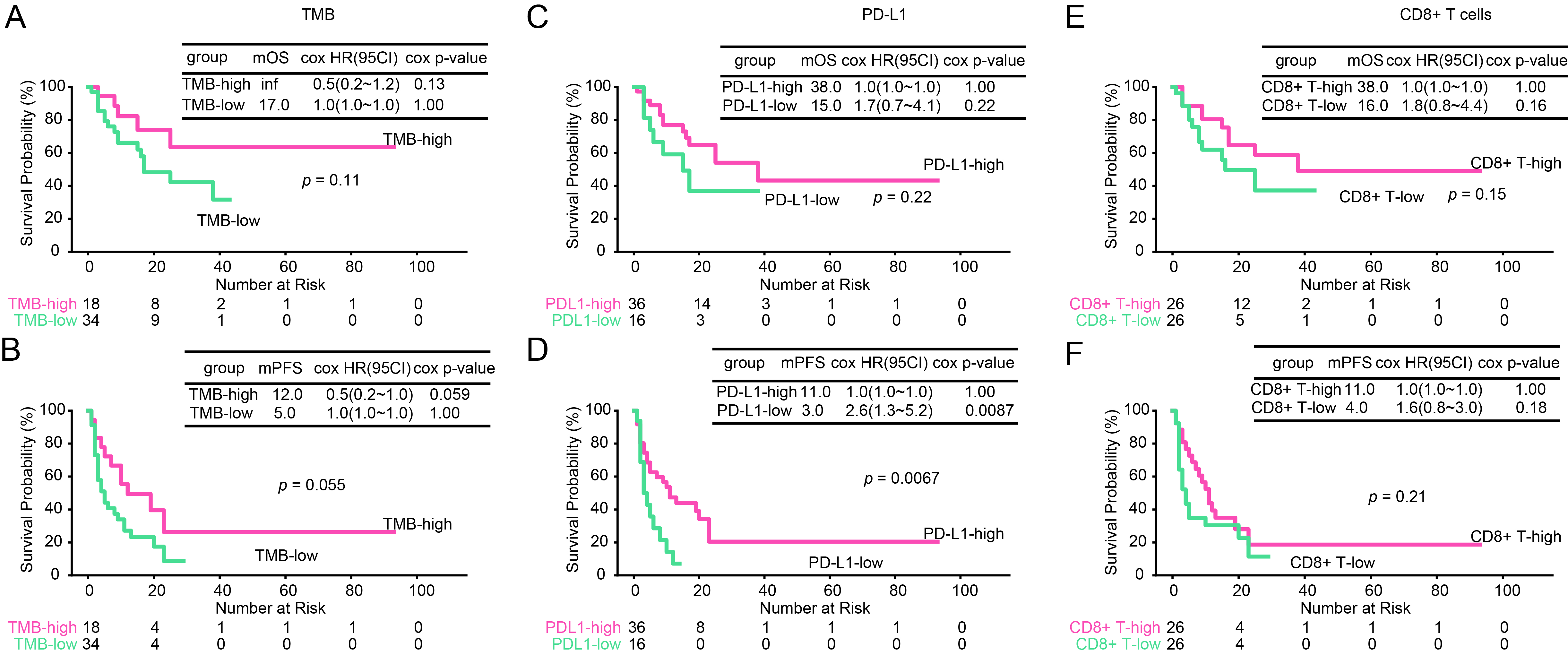

Supplement: Supplementary file 5 [file Image1.jpg]
